# Swainsonine Biosynthesis Genes in Diverse Symbiotic and Pathogenic Fungi

**DOI:** 10.1534/g3.117.041384

**Published:** 2017-04-03

**Authors:** Daniel Cook, Bruno G. G. Donzelli, Rebecca Creamer, Deana L. Baucom, Dale R. Gardner, Juan Pan, Neil Moore, Stuart B. Krasnoff, Jerzy W. Jaromczyk, Christopher L. Schardl

**Affiliations:** * Poisonous Plant Research Laboratory, United States Department of Agriculture–Agricultural Research Service, Logan, Utah 84321; † School of Integrative Plant Science, Cornell University, Ithaca, New York 14853; ‡ Department of Entomology, Plant Pathology, and Weed Science, New Mexico State University, Las Cruces, New Mexico 88001; § Department of Plant Pathology, University of Kentucky, Lexington, Kentucky 40546; ** Department of Computer Science, University of Kentucky, Lexington, Kentucky 40506; †† Robert W. Holley Center for Agriculture and Health, United States Department of Agriculture–Agricultural Research Service, Ithaca, New York 14853

**Keywords:** comparative genomics, dermatophytes, locoweed endophyte, pathogenic fungi, symbiotic fungi

## Abstract

Swainsonine—a cytotoxic fungal alkaloid and a potential cancer therapy drug—is produced by the insect pathogen and plant symbiont *Metarhizium robertsii*, the clover pathogen *Slafractonia leguminicola*, locoweed symbionts belonging to *Alternaria* sect. *Undifilum*, and a recently discovered morning glory symbiont belonging to order Chaetothyriales. Genome sequence analyses revealed that these fungi share orthologous gene clusters, designated “*SWN*,” which included a multifunctional *swnK* gene comprising predicted adenylylation and acyltransferase domains with their associated thiolation domains, a β-ketoacyl synthase domain, and two reductase domains. The role of *swnK* was demonstrated by inactivating it in *M. robertsii* through homologous gene replacement to give a ∆*swnK* mutant that produced no detectable swainsonine, then complementing the mutant with the wild-type gene to restore swainsonine biosynthesis. Other *SWN* cluster genes were predicted to encode two putative hydroxylases and two reductases, as expected to complete biosynthesis of swainsonine from the predicted SwnK product. *SWN* gene clusters were identified in six out of seven sequenced genomes of *Metarhzium* species, and in all 15 sequenced genomes of Arthrodermataceae, a family of fungi that cause athlete’s foot and ringworm diseases in humans and other mammals. Representative isolates of all of these species were cultured, and all *Metarhizium* spp. with *SWN* clusters, as well as all but one of the Arthrodermataceae, produced swainsonine. These results suggest a new biosynthetic hypothesis for this alkaloid, extending the known taxonomic breadth of swainsonine producers to at least four orders of Ascomycota, and suggest that swainsonine has roles in mutualistic symbioses and diseases of plants and animals.

Swainsonine is known to be produced by fungal symbionts of plants (endophytes), plant pathogens, and insect pathogens, but has not previously been reported from pathogens of humans and other mammals (Figure 1). This indolizidine alkaloid—a mannofuranose analog—specifically inhibits α-mannosidase II in the Golgi apparatus, disrupting the endomembrane system of the cell (Dorling *et al.* 1980; Tulsiani *et al.* 1982; Winchester *et al.* 1993), and is under consideration as a component of chemotherapeutic treatments for some cancers (Santos *et al.* 2011; Li *et al.* 2012). Swainsonine-producing endophytes belonging to *Alternaria* sect. *Undifilum* (Braun *et al.* 2003) can occur in certain legumes in the related genera *Astragalus*, *Oxytropis* (“locoweeds”), and *Swainsona*, in semiarid regions of Asia, the Americas, and Australia. Wildlife and livestock that feed on these plants can exhibit toxicosis (“locoism” or “pea struck”) characterized by weight loss, altered behavior, depression, decreased libido, infertility, abortion, birth defects, and death. Swainsonine is also produced by a recently discovered endophyte of the morning glory species, *Ipomoea carnea* (Cook *et al.* 2013). This *I. carnea* endophyte (ICE) has phylogenetic affinity to the order Chaetothyriales, but is of an undescribed species. Swainsonine is also produced by diverse fungi with other ecological functions; namely, the plant pathogen, *Slafractonia leguminicola* (Alhawatema *et al.* 2015), and the root-associated insect pathogen, *Metarhizium robertsii*, which is commonly used for organic plant protection (St. Leger and Wang 2010). Here, we identify and demonstrate function of orthologous swainsonine biosynthesis gene clusters (*SWN*) in these and other fungi, including all available members of the Arthrodermataceae—a family of dermatophytic human and animal pathogens.

**Figure 1:**
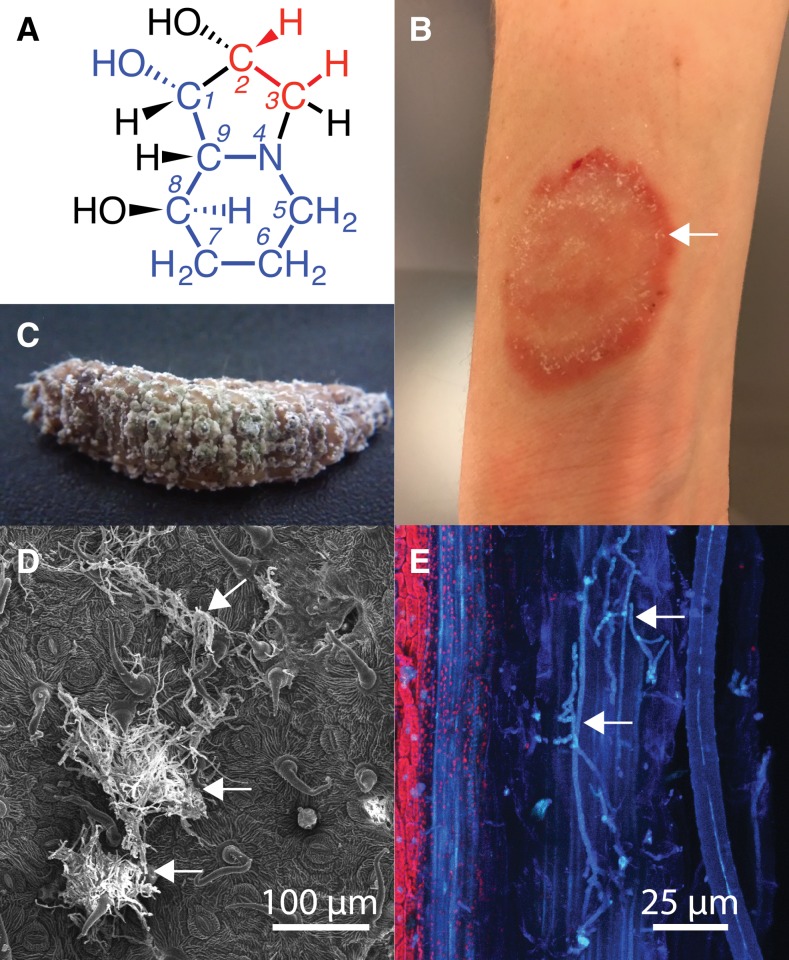
Swainsonine and swainsonine producers in their natural environments. (A) Structure of the indolizidine alkaloid, swainsonine. Atoms indicated in blue are known or predicted to be derived from pipecolic acid, and those in red from mevalonic acid. (B) Clinical symptoms of ringworm caused by *Trichophyton benhamiae* (Nenoff *et al.* 2014) (photograph provided by Dr. Pietro Nenoff, Laboratory for Medical Microbiology, Mölbis, Germany, and Dr. Ina Schulze, Markkleeberg near Leipzig, Germany). (C) Insect larva mummified by *Metarhizium* sp. White fungal mycelium is visible over the surface of the larva. (D) Scanning electron micrograph of ICE on the adaxial leaf surface. Arrows show masses of fungal hyphae (micrograph from Aziza Noor, New Mexico State University). (E) Confocal micrograph of endobiotic *A. oxytropis* (micrograph from Aziza Noor). Arrows indicate endobiotic hyphae.

## Materials and Methods

### Biological materials

The source and culture conditions for *Alternaria oxytropis* (=*Undifilum oxytropis*) are described in Reyna *et al.* (2012). The source and culturing conditions for the *Ipomoea carnea* endophyte (ICE) are described in Cook *et al.* (2013). *Slafractonia leguminicola* (=*Rhizoctonia leguminicola*) ATCC 26280 was obtained from the American Type Culture Collection (ATCC), and cultured as described by Alhawatema *et al.* (2015). All *Metarhizium* species were obtained from the ARSEF Collection of Entomopathogenic Fungi. All the dermatophytes were obtained from ATCC with the exception of *Trichophyton rubrum*, which was obtained from Theodore C. White at the University of Missouri-Kansas City.

### Genome sequencing and analysis

Fungal DNA was prepared using the ZR Fungal/Bacterial DNA MiniPrep kit (Zymo Research, Irvine, CA). Genome sequencing and assembly was performed at the Advanced Genetic Technologies Center (AGTC) of the University of Kentucky. The ICE genome was sequenced by pyrosequencing (Roche Diagnostics/454 Life Sciences Corp.) of sheared DNA fragments. A total of 1,334,638 pyrosequencing reads gave 995,708,063 bases, of which Newbler 2.8 (Roche Diagnostics/454 Life Sciences Corp.) aligned 1,284,087 reads totaling 949,835,583 aligned bases, to give an assembly of 32,767,887 bp in 307 contigs, with *N*50 = 380,768 bp.

Genomes of *A. oxytropis* and *S. leguminicola* were sequenced on the MiSeq platform (Illumina, San Diego, CA). For *A. oxytropis* a total of 40,814,896 paired MiSeq reads gave 8,826,667,915 bases, of which CLC Genomics Workbench 8.0.2 (Qiagen, Valencia, CA) matched 39,668,025 reads totaling 8,584,276,934 aligned bases, and paired 25,326,112 reads with an average paired read length = 426 bp, to give a genome assembly of 112,671,691 bp in 57,645 scaffolds, with *N*50 = 3841 bp. For *S. leguminicola*, a total of 27,839,555 paired MiSeq reads gave 6,027,678,056 bases, of which CLC Genomics Workbench 8.0.2 (Qiagen) aligned 27,189,730 reads totaling 5,880,240,986 aligned bases, and paired 25,491,776 reads with an average paired read length = 228 bp, to give a genome assembly of 49,495,572 bp in 24,662 scaffolds, with *N*50 = 18,922 bp.

### Chemical analysis

All isolates were grown on potato dextrose agar and were inoculated from an actively growing culture at a single point, and grown for 14 d in the dark. The *Metarhizium* species were grown at 25°, and the dermatophytes were grown at 29°. Cultures were air-dried and extracted with 2% acetic acid. Swainsonine was analyzed by LC-MS using methods described by Gardner and Cook (2016).

### Genetic manipulations of Metarhizium robertsii

**Figure 2:**
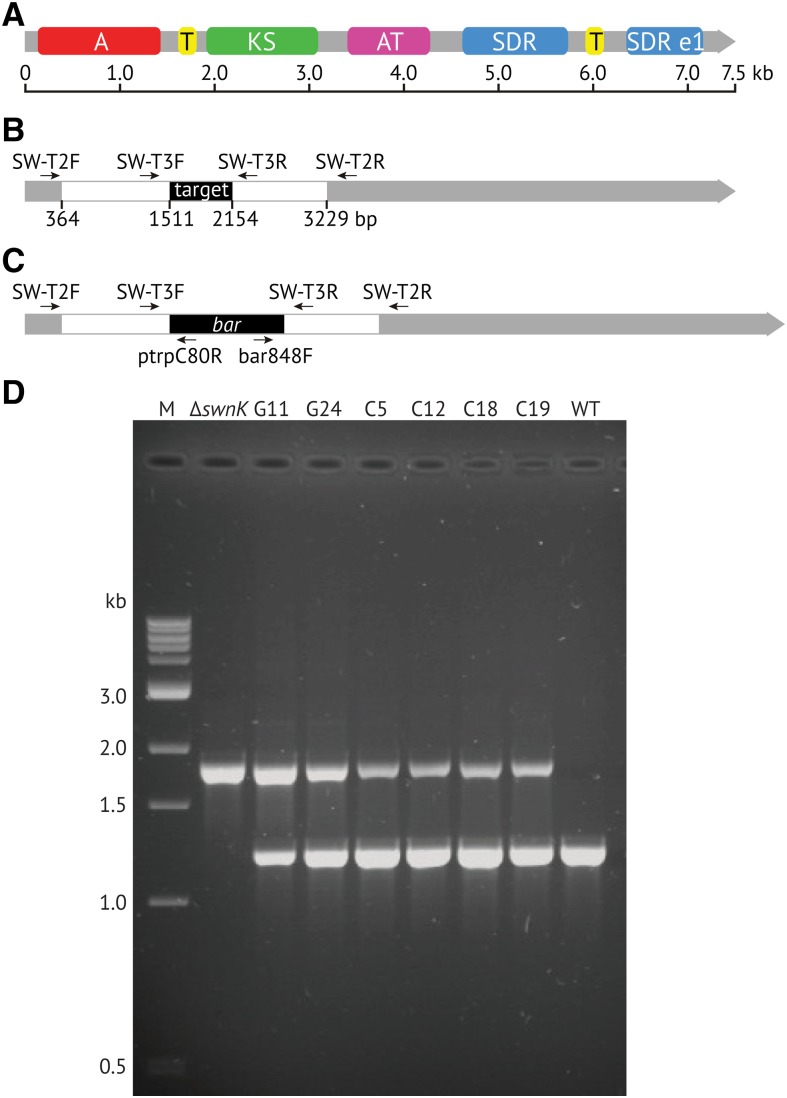
Tests for *swnK* gene knockouts and complemented strains of *M. robertsii* ARSEF 2575. (A) Domain structure of SwnK (see text and caption of Figure 3) mapped over the length of the gene. (B) Scale map of sequences cloned into the double crossover gene replacement construct (white), which flank the segment targeted for replacement (black). (C) Map of the ∆swnK mutation in which the target region is replaced with the bar gene. Labeled arrows indicate primers used for PCR screens (see Table S3 in File S1). (D) Electrophoretic analysis of PCR products generated with primers SW-T3F and SW-T3R and template DNAs from *∆swnK*, ectopic integrant controls G11 and G24, *∆swnK/swnK* complemented strains C5, C12, C18, and C19, and untransformed wild type (WT). PCR on intact and bar-disrupted loci is expected to generate products of 1240 and 1850 bp, respectively. Lane M contains molecular size markers.

A double crossover gene replacement construct (Figure 2), targeting the *swnK* gene, was assembled using two gene-specific DNA fragments (flank A and flank B) intercalated by the *bar* selection marker, which confers resistance to glufosinate ammonium (Donzelli *et al.* 2016). Gene-specific DNA fragments were produced by standard PCR reactions using primers listed in Supplemental Material, Table S3 in File S1, and *M. robertsii* ARSEF 2575 genomic DNA as the template. The *bar* selection marker was amplified from the pBARKS1 derivative pUCAP*bar*NOSII (Pall and Brunelli 1993; Donzelli *et al.* 2012) using primers indicated in Figure 2 and Table S3 in File S1. These three fragments were assembled into pBDU vector by the USER method (Nour-Eldin *et al.* 2006; Geu-Flores *et al.* 2007; Donzelli *et al.* 2012). A *swnK* complementation vector was produced by cloning a 9277 bp PCR product that included 1675 bp of the *swnK* promoter region, the entire *swnK* coding region (7467 bp) and 121 bp of the 3′ UTR region, into pBDUN binary vector. The pBDUN vector is a pPK2 (Covert *et al.* 2001) derivative carrying the nourseothricin resistance gene driven by the *Aspergillus nidulans trpC* promoter and compatible with the USER cloning method. Both gene replacement and gene complementation vectors were mobilized into *Agrobacterium tumefaciens* EHA105 by electroporation. *Agrobacterium tumefaciens*-mediated transformation of *M. robertsii* ARSEF 2575 was conducted as described (Moon *et al.* 2008) with the modification that selection of complemented transformants was with nourseothricin at 500 mg/liter.

Identification of *M. robertsii* ARSEF 2575 transformants carrying the *bar* gene at the targeted locus was conducted by PCR using primers SW-T2F and SW-T2R, annealing immediately outside the targeted region, in conjunction with primers ptrpc80R and bar848F, annealing within the *bar* selection cassette (Figure 2 and Table S3 in File S1). Single conidial progenies derived from putative homologous integrants were tested by PCR with primers SW-T3F and SW-T3R for integration of the complementation fragment in transformants displaying resistance to both glufosinate ammonium and nourseothricin.

### Virulence tests

Assays were conducted on *Drosophila suzukii* flies. Conidia (spores) of each respective *M. robertsii* strain were suspended in water with 0.01% Silwet L77 to give 2 ☓ 10^7^ conidia/ml. Batches of 48 adult females were dipped in the conidial suspension, and then incubated at 25°, 15 hr:9 hr light:dark cycle for 10 d. Treated insects were provided with preservative-free diet which was replaced every 24–48 hr. Differences in survival rates among treatments were quantified using survival analysis and Cox’s proportional hazards in JMP PRO 11 (SAS Institute Inc., Cary, NC).

### Data availability

Biological materials are available upon request. Figure S1 in File S1 shows the survival curves generated from two independent virulence assays where *D. suzukii* was used as the insect host. Table S1 in File S1 lists *SWN* gene homologs and swainsonine production in fungal cultures. Table S2 in File S1 shows results of assays for effects of swainsonine on virulence of *M. robertsii* on *D. suzukii*. Table S3 in File S1 shows sequences of oligonucleotide primers used in this study. Sequence data are available at GenBank and the accession numbers are listed in Table S1 in File S1.

## Results

Swainsonine precursors include pipecolic acid (Guengerich *et al.* 1973), an analog of the amino acid proline, and mevalonic acid (Clevenstine *et al.* 1979), a common building block for polyketides. Therefore, we hypothesized that a multifunctional enzyme with an amino-acid adenylylation (A) domain, an acyltransferase domain (AT), a β-ketoacyl synthase (KS) domain, and two phosphopantetheine-binding/thiolation domains (T) would catalyze the condensation of these two precursors. Based on studies of Harris *et al.* (1988a,b) with isotopically labeled precursors, we expected that additional domains or enzymes would be required for several reduction steps and two hydroxylations. Considering that genes for any particular specialized (secondary) metabolite tend to be closely linked in genomes of filamentous fungi (Spatafora and Bushley 2015), we used comparative genomics to identify orthologous clusters of genes for appropriate biosynthetic enzymes in the known swainsonine-producing fungi.

**Figure 3:**
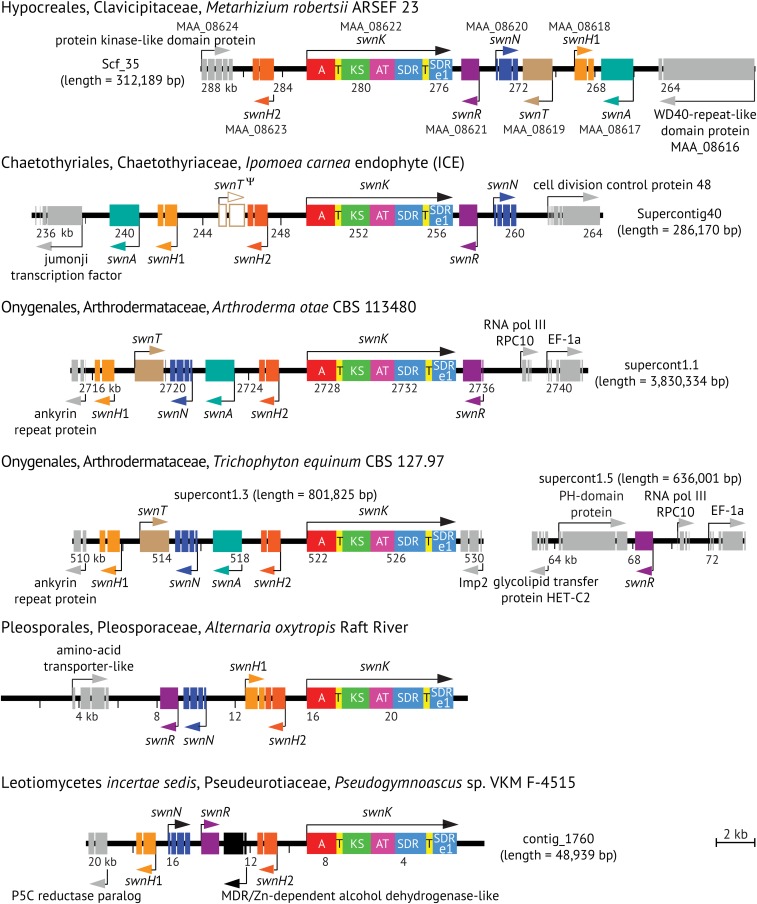
Structures of *SWN* gene clusters in representatives of five orders of fungi with diverse ecological roles. Taxa are listed by order, family, genus, species, and strain, and the scale on each map indicates the position of the gene cluster on the scaffold (supercontig) or contig indicated. Predicted functions of the gene products are: SwnA, an aromatic amino transferase; SwnH1 and SwnH2, 2-oxoglutarate- and Fe(II)-dependent dioxygenases; SwnN, an NmrA-like, NADB Rossmann-fold reductase; SwnR, an NADB Rossmann-fold reductase; SwnT, a transmembrane transporter; and SwnK, a multifunctional protein with adenylylation (A), phosphopantetheine-binding/thiolation (T), β-ketoacyl synthase (KS), acyltransferase (AT), reductase (SDR), and thioester reductase (SDR e1) domains. *SWN* genes and *swnK* domains are color-coded, flanking genes are shown in gray, and *swnT* of ICE is represented as a likely pseudogene.

The genome of ICE (Cook *et al.* 2013) was sequenced and annotated to model its genes. Inferred protein sequences from this genome and the published genome of *M. robertsii* ARSEF 23 (Gao *et al.* 2011) were searched by hidden Markov models (HMM) with InterProScan to identify putative functional domains. The sequences were also submitted as BLASTp queries against fungal protein sequences in the nonredundant GenBank database. Results were displayed in a modified GBrowse version 1.70 for visual inspection to identify any gene models with similar structures that included A, AT, KS, and T domains, which were then compared by reciprocal best-BLASTp between the two genomes. The results indicated that ICE and *M. robertsii* shared orthologous candidate genes, one of which, with an appropriate multi-domain structure, was designated *swnK* (Figure 3). Genes closely linked with *swnK* were also inspected for putative functions, and compared by best-BLASTp analysis to identify shared genes of the putative swainsonine-biosynthesis gene cluster, designated *SWN* (GenBank accessions KY365740 and JELW01000031.1). In addition to *swnK*, the *SWN* clusters included *swnN* and *swnR*, putatively encoding reductases of different Rossmann-fold families, and *swnH*1 and *swnH*2, putatively encoding related 2-oxoglutarate-dependent nonheme-iron dioxygenases. There were no widely shared regulatory genes in the orthologous *SWN* clusters, although several of the clusters have adjacent genes that may be regulatory.

As a definitive test of the role of *swnK* in swainsonine biosynthesis, we disrupted this gene in *M. robertsii* ARSEF 2575 by introducing the selectable *bar* gene for bialaphos resistance in place of a segment that included the T domain adjacent to the A domain (Figure 2). The resulting ∆*swnK* mutant failed to produce swainsonine at detectable levels. This mutant was then complemented by reintroduction of wild-type *swnK*, restoring swainsonine production in several independent transformants (Table 1). Most of the complemented transformants produced higher levels of swainsonine in culture than did the wild-type strain.

**Table 1:** Results of molecular genetic tests for the role of *swnK* in swainsonine biosynthesis

Strain	Genotype	Comment	Swainsonine Concentration (µg/g Dry Mass ± SD)
ARSEF 2575	WT	Wild-type strain	653 ± 119
∆*swnK*	∆*swnK*	*swnK* disruption mutant	Not detected
G11	WT + *bar*	Ectopic transformant	620 ± 103
G24	WT + *bar*	Ectopic transformant	633 ± 81
C5	*∆swnK + swnK*	Complemented mutant	1268 ± 470
C12	*∆swnK + swnK*	Complemented mutant	1141 ± 497
C18	*∆swnK + swnK*	Complemented mutant	145 ± 46
C19	*∆swnK + swnK*	Complemented mutant	2573 ± 1408

Exhaustive BLASTp and tBLASTn searches of published genome sequences revealed *SWN*-cluster orthologs in five different orders of filamentous Ascomycota (Figure 3 and Table S1 in File S1). These included the Arthrodermataceae (order Onygenales), which are skin pathogens that cause ringworm and athlete’s foot in humans and other mammals. All Arthrodermataceae possessed *swnH*1, *swnH*2, *swnK*, *swnN*, and *swnR*, but in the *Trichophyton* species *swnR* was present in a separate locus (Figure 3 and Table S1 in File S1). In two of the *Trichophyton* genome sequences, *swnR* was not annotated, but their apparently complete *swnR* gene orthologs could be identified by tBLASTn. (These are listed in Table S1 in File S1 by their nucleotide sequence accessions AOKT01000327.1 and LHPM01000018.1.) Representatives of six species of Arthrodermataceae—namely, *Arthroderma otae* (=*Microsporum canis*), *Nannizzia gypsea* (=*Microsporum gypseum*), *Trichophyton benhamiae* (=*Arthroderma benhamiae*), *Trichophyton interdigitale*, *Trichophyton equinum* (=*Trichophyton tonsurans*), and *Trichophyton rubrum*—were cultured and tested for swainsonine, and all except *Ar. otae* produced the alkaloid. Considering that symbiotic fungi such as *Epichloë* species tend to express alkaloids only in their hosts and not in culture (Chujo and Scott 2014), it is possible that *Ar. otae* produces swainsonine only in its host, or that it requires other culture conditions. *SWN* clusters were also identified in all sequenced isolates of *Metarhizium* species except *Metarhizium album* ARSEF 1941. Cultures of all eight available isolates of six *Metarhizium* species, but not of *M. album*, produced detectable levels of swainsonine (Table S1 in File S1), showing a strong correlation of *SWN* cluster presence and swainsonine production.

Whole genome shotgun sequencing was also conducted on the genomes of the clover black patch pathogen, *Slafractonia leguminicola* (Alhawatema *et al.* 2015), and the endophyte, *Alternaria oxytropis* (Pryor *et al.* 2009), both of which are known swainsonine producers in the order Pleosporales. Putative orthologs of *swnK*, *swnN*, *swnR*, *swnH*1, and *swnH*2 were identified in both, but not necessarily on shared scaffolds. In the *A. oxytropis* genome assembly, the apparent orthologs were on three scaffolds, but scaffold ends overlapped within coding sequences of *swnN* and *swnH*1 to permit manual assembly of the entire cluster (Figure 3; GenBank accession KY365741). For *S. leguminicola* (GenBank accessions KY365742–KY365746), *swnK*, *swnN*, *swnR*, and *swnT* assembled uniquely and apparently completely on separate scaffolds, *swnH*1 and *swnH*2 assembled together in a scaffold as convergently transcribed genes, similar to the arrangement in *A. oxytropis*. Therefore, there was no evidence for or against clustering of *SWN* genes in *S. leguminicola*. Also like *A. oxytropis*, no *swnA* ortholog was found in *S. leguminicola*.

*S. leguminicola* produces two distinct indolizidines, swainsonine and slaframine, of which the latter causes slobbers in livestock that graze infected legume foliage (Harris *et al.* 1988b; Croom *et al.* 1995). Given the structural similarity of swainsonine and slaframine, a second *swnK* homolog was expected in *S. leguminicola*. In fact, two *swnK* paralogs were identified (GenBank accessions KY365747 and KY365748), both encoding proteins with the SwnK domain structure. The paralogs had similar levels of divergence from each other and from SwnK, with 53.1–55.4% identity to SwnK of *S. leguminicola*, *A. oxytropis*, *M. robertsii*, and ICE. In contrast, SwnK orthologs all shared >70% identity with each other. Presumably one SwnK paralog is involved in biosynthesis of slaframine, and conceivably the other is involved in biosynthesis of a related alkaloid yet to be identified.

The genes designated *swnA* and *swnT* were present in *SWN* clusters of some but not all swainsonine producers. (Figure 3 and Table S1 in File S1). SwnA was a putative aminotransferase, and SwnT was related to a transmembrane choline transporter. Orthologs of *swnT* were present in all except the two endophytes, ICE, which had a *swnT* pseudogene, and *A. oxytropis*, which completely lacked *swnT*. It is noteworthy that an orthologous *swnT* gene was present in *S. leguminicola*, which is in the same order as *A. oxytropis*, indicating that the gene was lost in an ancestor of the endophyte *A. oxytropis*. The possibility deserves further investigation that loss of *swnT* is related to evolution of mutualistic symbionts, perhaps by altering the localization of swainsonine.

BLASTp searches of sequenced genomes also revealed gene clusters orthologous to *SWN* in *Pseudogymnoascus* sp. VKM F-4515 from permafrost soil (Figure 3), and in a recently published genome from a fungus designated fungal sp. No.14919 (Table S1 in File S1). As in the Arthrodermataceae, *Metarhizium* species, *A. oxytropis* and ICE, these species had apparent *swnK* orthologs closely linked to *swnH*1, *swnH*2, *swnN*, and *swnR*. In fungal sp. No.14919 the arrangement of these genes, plus a *swnT* ortholog, was similar to that in *Metarhizium robertsii* ARSEF 23 (Figure 3). *Pseudogymnoascus* sp. VKM F-4515 and fungal sp. No.14919 had distinct additional genes for putative biosynthetic enzymes adjacent to the cluster. It seems likely that these two species also produce swainsonine or a related metabolite.

We employed ∆*swnK*, *swnK*-complemented strains, and wild-type *M. robertsii* to test the role of swainsonine in pathogenicity to the insect, *D. suzukii*. Survivorship curves indicated no significant reduction in virulence for the ∆*swnK* strain (Figure S1 and Table S2 in File S1). In fact, in most tests swainsonine-producing strains exhibited slightly less virulence than did the knockout strain.

## Discussion

**Figure 4:**
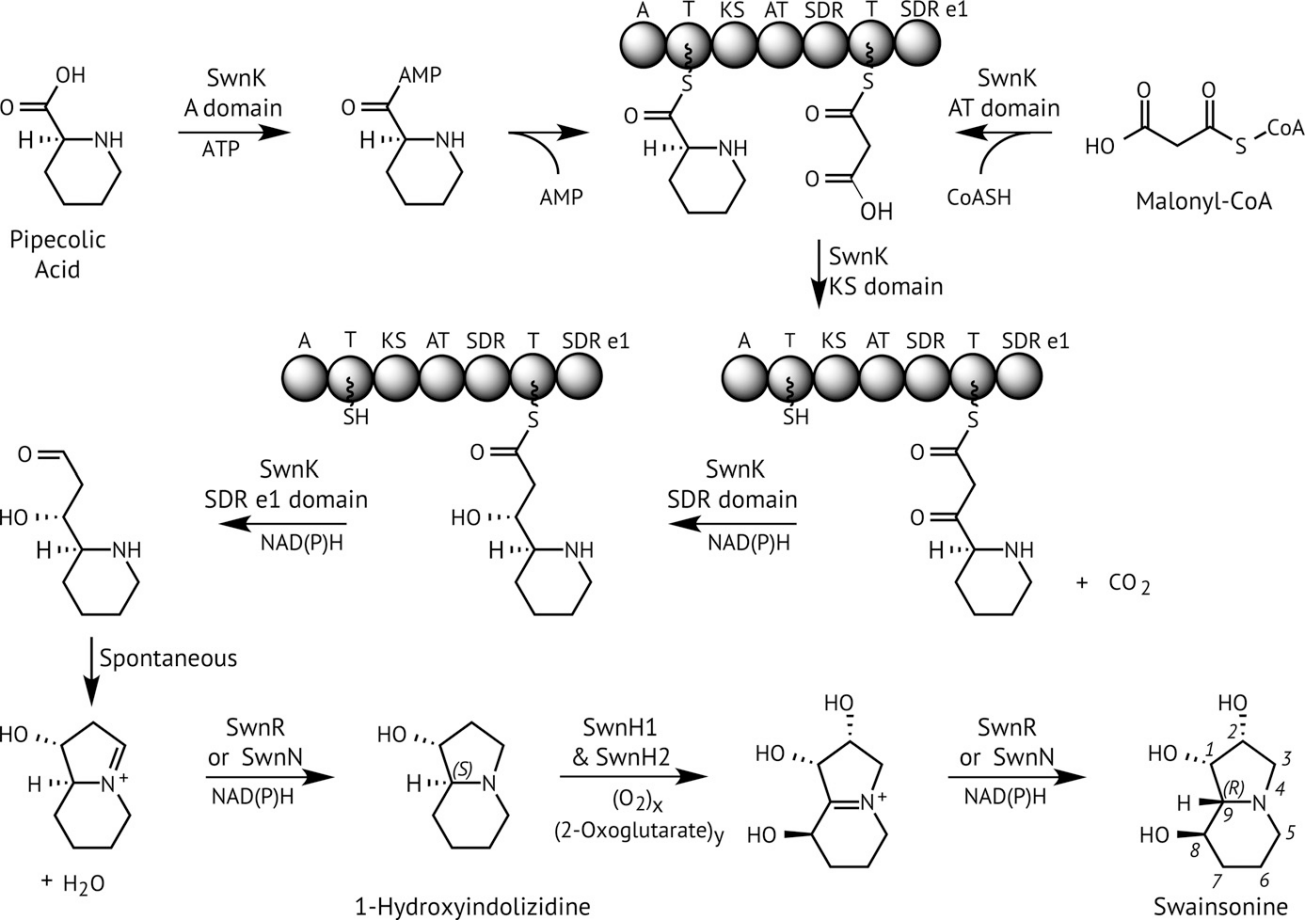
Proposed swainsonine biosynthetic pathway based on predicted gene functions. Predicted functions of SwnK domains and other enzymes are listed in the caption of Figure 3.

Our comparative genomic analysis proved fruitful in revealing a gene cluster that was common among swainsonine producers, and that included a ketide synthase gene (which we designated *swnK*) with appropriate domains for the first steps of swainsonine biosynthesis. Subsequent inactivation of *swnK* in *M. robertsii*, followed by complementation of the ∆*swnK* mutant, provided for confirmation that this gene was required for swainsonine production. The arrangement of domains in the inferred SwnK polypeptide sequence, together with published results of isotope labeling experiments (Harris *et al.* 1988b), suggested biosynthetic roles for SwnK, as well as for the SwnR and SwnN reductases, and the SwnH1 and SwnH2 hydroxylases encoded by nearby genes (Figure 4). Based on the presence and positions in SwnK of the two reductase domains, SDR (β-ketoacyl reductase) and SDR e1 (thioester reductase) (Figure 2 and Figure 4), we predict that the intermediate released from this enzyme has a hydroxyl group instead of the keto group proposed by Harris *et al.* (1988b). This intermediate should cyclize to generate a C3=N^+^4 iminium ion that we propose to be reduced by the action of SwnR or SwnN, giving 1-hydroxyindolizidine, which has been reported from the locoweed species, *Astragalus oxyphysus* (Harris *et al.* 1988a). Subsequent oxygenations must occur at carbons 2 and 8, consistent with predicted activities of SwnH1 and SwnH2. Furthermore, Harris *et al.* (1988b) present strong evidence for epimerization at carbon 9, suggesting a second iminium ion intermediate, C9=N^+^4, perhaps generated by the action of SwnH1 or SwnH2. Thus, the presence of the two putative reductase genes, *swnN* and *swnR*, is consistent with a requirement to reduce both a C3=N^+^4 and a C9=N^+^4 iminium ion in the proposed pathway.

The diversity of fungi that produce swainsonine suggests multiple ecological roles for the alkaloid among symbionts and parasites of plants and animals. So far, indications have been surprising. In the case of locoweeds, grazing mammals can develop a preference despite the devastating cytotoxic and neurological effects of swainsonine (Ralphs *et al.* 1990; Pfister *et al.* 2003). Thus, the alkaloid does not seem to deter these herbivores. Also, our tests of *M. robertsii* on the insect model, *D. suzukii*, indicate that swainsonine does not contribute, and may actually moderate, virulence. It is possible that swainsonine may have a deterrent or toxic effect on other herbivores or other microbes, and that it may have a role in maintaining some stable symbioses of fungi with host plants. Furthermore, the ubiquity of *SWN* genes in the Arthrodermataceae raises the possibility that swainsonine has a role in the etiology of skin infections by these dermatophytic fungi—an important consideration for veterinary and human medicine.

## Supplementary Material

Supplemental material is available online at www.g3journal.org/lookup/suppl/doi:10.1534/g3.117.041384/-/DC1.
