# Supplementary material for: Swainsonine Biosynthesis Genes in Diverse Symbiotic and Pathogenic Fungi
Source: G3 (Bethesda). 2017 Apr 3;7(6):1791–7. doi: 10.1534/g3.117.041384 (PMC5473758; doi:10.1534/g3.117.041384)
Supplement: Supplementary file 1 [file 1791File001.pdf]

**Supplemental Materials:**

Figure S1. Survival curves generated from two independent virulence assays where *Drosophila suzukii* was used as the insect host.

Table S1. *SWN* gene homologues and swainsonine production in fungal cultures.

Table S2. Results of assays for effects of swainsonine on virulence of *Metarhizium robertsii* on *Drosophila suzukii*.

Table S3. Oligonucleotide primers used in this study.

**Table S1.** *SWN* gene homologues and swainsonine production in fungal cultures.

| Taxon and strain | Swainsonine production [b] | GenBank accessions [a] | | | | | | |
|---|---|---|---|---|---|---|---|---|
| | | ***swnA*** | ***swnH*1** | ***swnH*2** | ***swnK*** | ***swnN*** | ***swnR*** | ***swnT*** |
| *Alternaria oxytropis* Raft River | + | – | KY365741 | KY365741 | KY365741 | KY365741 | KY365741 | – |
| *Arthroderma benhamiae* MYA-4681 CBS 112371 | + | XP_003014122 | XP_003014119 | XP_003014123 | XP_003014124 | XP_003014121 | XP_003016302 | XP_003014120 |
| *Arthroderma otae* (= *Microsporum canis*) MYA-4605 CBS 113480 | – | XP_002850893 EEQ28109 | XP_002850896 EEQ28112 | XP_002850892 EEQ28108 | XP_002850891 EEQ28107 | XP_002850894 EEQ28110 | XP_002850890 EEQ28106 | XP_002850895 EEQ28111 |
| Chaetothyrialean endophyte of *Ipomoea carnea* (ICE) | + | KY365740 | KY365740 | KY365740 | KY365740 | KY365740 | KY365740 | KY365740 [c] |
| Fungal sp. No.14919 | n.t. | – | GAW19807 | GAW19802 | GAW19803 | GAW19805 | GAW19804 | GAW19806 |
| *Metarhizium acridum* ARSEF 324 | + | s.n.a. | s.n.a. | s.n.a. | s.n.a. | s.n.a. | s.n.a. | s.n.a. |
| *Metarhizium acridum* ARSEF 3341 | + | s.n.a. | s.n.a. | s.n.a. | s.n.a. | s.n.a. | s.n.a. | s.n.a. |
| *Metarhizium acridum* CQMa 102 | n.t. | XP_007815894 | XP_007815893 | XP_007815888 | XP_007815889 | XP_007815891 | XP_007815890 | XP_007815892 |

| | | | | | | | | |
|---|---|---|---|---|---|---|---|---|
| *Metarhizium album* ARSEF 1941 | – | – | – | – | – | – | – | – |
| *Metarhizium anisopliae* BRIP 53284 | n.t. | (KJK92562) | KJK85334 | KJK85339 | KJK85338 | KJK85336 | KJK85337 | KJK85335 |
| *Metarhizium anisopliae* BRIP 53293 | n.t. | (KJK74447) | KJK74448 | KJK74453 | KJK74452 | KJK74450 | KJK74451 | KJK74449 |
| *Metarhizium anisopliae* ARSEF 549 | + | KID61013 | KID61012 | KID61007 | KID61008 | KID61010 | KID61009 | KID61011 |
| *Metarhizium anisopliae* E6 | n.t. | – | KFG82168 | KFG82173 | KFG82172 | KFG82170 | KFG82171 | KFG82169 |
| *Metarhizium brunneum* ARSEF 3297 | + | XP_014543161 | XP_014543162 | XP_014543167 | XP_014543166 | XP_014543164 | XP_014543165 | XP_014543163 |
| *Metarhizium guizhouense* ARSEF 977 | tr | KID83598 | KID83599 | KID83604 | KID83603 | KID83601 | KID83602 | KID83600 |
| *Metarhizium majus* ARSEF 297 | tr | XP_014575317 | XP_014575316 | XP_014575311 | XP_014575312 | XP_014575314 | XP_014575313 | XP_014575315 |
| *Metarhizium robertsii* ARSEF 23 | + | XP_007824806.2 | XP_007824807 | XP_007824812 | XP_007824811 | XP_007824809 | XP_007824810 | XP_007824808 |
| *Metarhizium robertsii* ARSEF 2575 | + | EXU97977 | EXU97978 | EXU97983 | EXU97982 | EXU97980 | EXU97981 | EXU97979 |

| | | | | | | | | |
|---|---|---|---|---|---|---|---|---|
| *Nannizzia gypsea* CBS 118893 (= *Microsporum gypseum*) MYA-4604 | + | XP_003176905 | XP_003176902 | XP_003176906 | XP_003176907 | XP_003176904 | XP_003176908 | XP_003176903 |
| *Pseudogymnoascus* sp. VKM F-4515 (FW-2607) | n.t. | – | KFY51104 | KFY51100 | KFY51099 | KFY51103 | KFY51102 | – |
| *Slafractonia leguminicola* ATCC 26280 | + | – | KY365745 | KY365745 | KY365746 | KY365744 | KY365743 | KY365742 |
| *Trichophyton interdigitale* MYA-3108 tinea pedis H6 | + | EZF30504 | EZF30351 | EZF30503 | EZF30502 | EZF30348 | EZF32734 | EZF30349 |
| *Trichophyton equinum* CBS 127.97 (= *Trichophyton tonsurans*) MYA-4606 | + | EGE01984 | EGE01987 | EGE01983 | EGE01982 | EGE01985 | EGE02341 | EGE01986 |
| *Trichophyton rubrum* MYA-4607 CBS 118892 | tr | XP_003238868 | XP_003238865 | XP_003238869 | XP_003238870 | XP_003238867 | XP_003238559 | XP_003238866 |
| *Trichophyton interdigitale* MR816 | n.t. | KDB22224 | KDB22220 | KDB22225 | KDB22226 | KDB22223 | KDB22462 | KDB22222 |
| *Trichophyton rubrum* CMCC(F)T1i | n.t. | OAL63128 | OAL63131 | OAL63127 | OAL63126 | OAL63129 | LHPM01000018 | OAL63130 |

| | | | | | | | | |
|---|---|---|---|---|---|---|---|---|
| *Trichophyton rubrum* CBS 289.86 | n.t. | EZF58568 | EZF58564 | EZF58569 | EZF58570 | EZF58567 | EZF48315 | EZF58566 |
| *Trichophyton rubrum* CBS 735.88 | n.t. | EZG01519 | EZG01515 | EZG01520 | EZG01521 | EZG01518 | EZG01771 | EZG01517 |
| *Trichophyton rubrum* CBS 100081 | n.t. | EZF37321 | EZF37322 | EZF37317 | EZF37323 | EZF37320 | AOKT01000327 | EZF37319 |
| *Trichophyton soudanense* CBS 452.61 | n.t. | EZF69146 | EZF69142 | EZF69147 | EZF69148 | EZF69154 | EZF69504 | EZF69144 |
| *Trichophyton tonsurans* CBS 112818 | n.t. | EGD97141 | EGD97144 | EGD97140 | EGD97139 | EGD97142 | EGD97826 | EGD97143 |
| *Trichophyton verrucosum* HKI 0517 | n.t. | XP_003020761 | XP_003020758 | XP_003020762 | XP_003020763 | XP_003020760 | XP_003022179 | XP_003020759 |
| *Trichophyton violaceum* CMCC(F)T3l | n.t. | OAL75149 | OAL75146 | OAL75150 | OAL75151 | OAL75148 | OAL69278 | OAL75147 |

[a] Genes are identified by GenBank accession numbers either for the predicted protein or the assembled genomic sequence bearing the genes. Symbols and abbreviations: – = not found, s.n.a. = sequence not available.

[b] Symbols and abbreviations: + = detected at > 10 µg/g, tr = trace detected at ≤ 10 µg/g, – = undetected, n.t. = not tested. Literature reports production of swainsonine by the respective strains of ICE (COOK *et al.* 2013) and *A. oxytropis* (GRUM *et al.* 2013), and by another isolate of *S. leguminicola* (HARRIS *et al.* 1988b).

[c] The *swnT* homologue in ICE is probably a pseudogene.

**Table S2.** Results of assays for effects of swainsonine on virulence of *Metarhizium robertsii* on *Drosophila suzukii*.

| Assay ID | Strain [a] | Swainsonine (Strain/WT) [b] | Mean Survival Time (days) | | Hypothesis test [c] | |
|---|---|---|---|---|---|---|
| | | | Reference (WT applied) | Strain applied [c] | Log-rank | Wilcoxon |
| 1 | Δ*swnK* | 0 | 6.90 | 6.09 | 0.096 | 0.187 |
| 2 | Δ*swnK* | 0 | 6.74 | **<u>5.75</u>** | **<u>0.007</u>** | **<u>0.010</u>** |
| 3 | Δ*swnK* | 0 | 6.11 | **7.21** | **0.001** | **0.001** |
| 1 | *swnK*-C18 | 0.25 | 6.90 | **7.60** | 0.054 | **0.011** |
| 2 | *swnK*-C18 | 0.25 | 6.74 | 6.60 | 0.517 | 0.660 |
| 1 | *swnK*-C5 | 2 | 6.90 | **7.72** | **<.0001** | **<.0001** |
| 2 | *swnK*-C5 | 2 | 6.74 | **8.30** | **<.0001** | **<.0001** |
| 3 | *swnK*-C5 | 2 | 6.11 | **7.71** | **<.0001** | **<.0001** |
| 3 | *swnK*-C12 | 2 | 6.11 | **8.71** | **<.0001** | **<.0001** |
| 3 | *swnK*-C19 | 4 | 6.11 | **6.93** | 0.061 | **0.005** |

[a] *Metarhizium robertsii* strain, where WT = wild type ARSEF 2575, Δ*swnK* = the *swnK* deletion mutant, and *swnK*-C18, *swnK*-C5, *swnK*-C12 and *swnK*-C19 are four independent transformants of Δ*swnK* with *swnK* from WT (complemented strains).

[b] Approximate ratio of swainsonine produced in cultures of the respective strain over swainsonine produced in cultures of wild-type *M. robertsii* ARSEF 23 (WT).

[c] Bold-face type indicates significant difference in mean survival times of insects treated with the respective mutant compared to those treated with WT. Underline indicates significant reduction in mean survival time compared to those treated with WT.

**Table S3.** Oligonucleotide primers used in this study.

| Name | Sequence (5` to >3`) [a] | Remarks |
|---|---|---|
| SWKO-AF | GGGAAAGdUGAAGATGCCGGGCTCAGACT | *swnK* knockout construct, flank A |
| SWKO-AR | ATCATCCdUGTTGACCTTTTCGCTGGGAGAC | *swnK* knockout construct, flank A |
| SWKO-BF | ACTTGTGGdUAACGATCCGGCTATACACGAG | *swnK* knockout construct, flank B |
| SWKO-BR | GGAGACAdUTCCGCTTGTGCCCTGAGAG | *swnK* knockout construct, flank B |
| BarExprSF | AGGATGAdUAGAAGATGATATTGAAGGA | *bar* cassette |
| BarExprSR NOS | ACCACAAGdUCATGTTTGACAGCTTATCAT | *bar* cassette |
| SW-T2F | CGCGCTCCATCGACCTCATT | Anneals outside the region targeted for homologous integration |
| SW-T2R | CATCACGCAAGGGGCTGTC | Anneals outside the region targeted for homologous integration |
| ptrpc80R | CCGCCTGGACGACTAAACC | Anneals at the 5` end of the *trpC* promoter of the *bar* expression cassette |
| bar848F | ACTGGCATGACGTGGGTTTCTGG | Anneals at the 3` end of the *bar* ORF |
| SW-T3F | TGATCGTCTGGTGGCGTATTGTGT | Anneals inside the targeted region. |
| SW-T3R | ATCGGACAGGCGCTTGAGAACTAT | Anneals inside the targeted region. |
| SW-CF | GGGAAAGdUTTTGAAGATGGGCTGGTG | complementation |
| SW-CR | GGAGACAdUCAGGCTTGGGAACAGAATGAGACC | complementation |

[a] dU = internal deoxyuracil, which is coded as /ideoxyU/ by Integrated DNA Technologies.

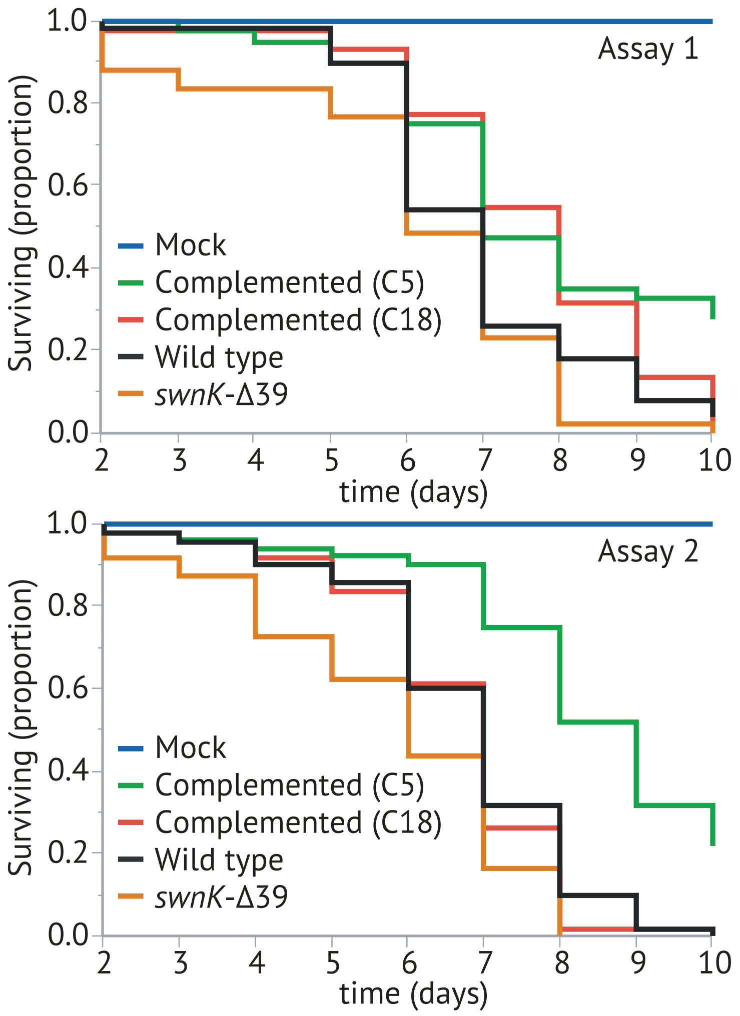


**Figure S1** Survival curves generated from two independent virulence assays where *Drosophila suzukii* was used as the insect host. Cox's proportional hazards presented here were generated using the wild type as the control and represent the increase of risk associated to the inoculation of a mutant compared to the WT. Thus, a CPH of 1 indicates no risk change compared to the WT; a CPH of 2 indicates a mortality risk twice that inflicted by the WT in the same assay conditions.
